# Development of Subcritical Water Extraction for Areca Alkaloids and Its Influence on the Structure of Areca Nut Husk

**DOI:** 10.3390/molecules30040886

**Published:** 2025-02-14

**Authors:** Hongliang Zhao, Jinghao Yang, Jun Zeng, Bosheng Zhou, Min Yang, Xiaohong Yang, Ranfeng Sun

**Affiliations:** 1 School of Life and Health Sciences, Hainan University, Haikou 570228, China; hnxxzhao@126.com (H.Z.); 17733131251@163.com (B.Z.); 990408@hainanu.edu.cn (X.Y.); 2 Hainan Academy of Inspection and Testing, Haikou 570203, China; 3 Institute of Tropical Bioscience and Biotechnology, Chinese Academy of Tropical Agricultural Sciences, Haikou 571199, China; yangjinghao@itbb.org.cn (J.Y.); zengjun@itbb.org.cn (J.Z.); 4 School of Pharmacy, Gannan Medical University, Ganzhou 341000, China

**Keywords:** subcritical water extraction, areca nut, alkaloid, husk, fiber

## Abstract

The optimal process parameters of subcritical water extraction (SWE) for areca alkaloids in areca nut (AN) husk were described: extraction temperature of 110.87 °C, liquid-to-solid ratio of 18.98:1 and extraction time of 50.01 min. It was found that the factors influencing the process in order of significance were extraction time > extraction temperature > liquid-to-solid ratio. Considering practical conditions, the parameters were adjusted to the extraction temperature of 110 °C, the liquid-to-solid ratio of 19:1 and the extraction time of 50 min. The measured extraction rate was 81.7%, which is close to the predicted value, indicating that the extraction process optimized by Response Surface Methodology (RSM) is feasible. Meanwhile, preliminary results from XRD and FT-IR indicated that SWE had a significant influence on the structure of the AN husk, possibly by damaging some of the crystalline regions of cellulose in the AN husk or reducing the concentration of various functional groups. Overall, this study provided valuable insights in the SWE for areca alkaloids and its influence on the structure of areca nut husk, and further exploration for industrialization is still under development in our laboratory.

## 1. Introduction

*Areca catechu* L. is a widely distributed tropical crop, which belongs to the arecaceaeh with a long history of being consumed in South and Southeast Asia [1]. Its fruit, areca nut (AN), can be used individually or together for consumption or medicinal purposes [2,3]. As an edible AN, the chewing is the most common means of intake, due to its unique flavor and chewing characteristics. Worldwide, there are over 600 million betel nut chewers, and this number is still growing [4]. It is estimated that about 10–20% of the global population consume AN, making it the fourth most consumed addictive substance after nicotine, alcohol, and caffeine [5]. Meanwhile, AN possesses a complex chemical composition [6], such as polyphenols (10–30%), polysaccharides (18–25%), fibers (10–15%), fatty acids (10–15%) and alkaloids (0.3–0.7%), and most of them are bioactive components. In this regard, AN has a long medicinal history, which is recorded in the Compendium of Materia Medica [7] and has been included in the Pharmacopoeia of China [8]. AN appears in many prescriptions to treat malaria, abdominal pain, periodontitis, etc. [9,10]. In addition, AN fiber has good physical properties and can be used as an alternative to synthetic fiber in textile and other industries [11,12]. Therefore, AN is a cash crop with rapid development. According to data released by the United Nations Food and Agriculture Organization, the total global production of AN in 2020 was 1,796,300 tons, with a harvested area of 1,226,100 ha. Among these, Hainan’s production of AN ranked second in the world. Data show that in 2016, the planting area of areca nut in Hainan Province reached 3.8 million mu, with a production value of over USD 40 billion [13]. By 2020, the planting area of areca nut had increased to 88,500 ha, with an annual output of 283,300 tons, making it the country with the highest yield per unit area in the world [14].

Generally, AN fruit consists of two main parts: the husk and kernel. The husk usually accounts for 60–80% of the mass or volume in the whole fresh AN, and it is primarily composed of cellulose (35–64.8%), hemicellulose, lignin (13–26%) and pectin (7%) [15,16]. Due to the high crude fiber content in AN husk, which gives it a hard and rough texture, chewing ANs easily causes long-term mechanical irritation to the oral mucosa. This is also a key risk factor for oral cancer [17,18]. In the AN processing industry, numerous AN husks are discarded once their nuts are separated, which results in the waste of biomass energy with potential application value [19,20]. Meanwhile, people who chew ANs tend to become addicted to them, mainly because of the presence of areca alkaloids. The areca alkaloids in AN are approximately 0.3–0.7%, and their structures are similar to that of nicotine [21]. Among them, four main alkaloids have been identified, namely arecoline (0.30–0.63%), arecaidine (0.31–0.66%), guvacoline (0.03–0.06%) and guvacine (0.19–0.72%). Areca alkaloids are important bioactive substances, which can affect the nervous system, cardiovascular system and digestive system, and have anthelmintic and antibacterial effects. In the broader field of AN research, studies on areca alkaloids contribute to the development of new medications, such as those for treating glaucoma or as pre-exercise supplements to enhance physical performance. Additionally, understanding the pharmacological properties of areca alkaloids is crucial for recognizing its potential risks and benefits to human health, especially considering the association between long-term AN chewing and oral cancer. In other words, research on areca alkaloids not only enhances the understanding of their biological activities but also provides a scientific basis for the prevention and treatment of related diseases. Although their content in AN husk is relatively low, it also has significant pharmacological effects and research value [6]. Therefore, extracting areca alkaloids from a large number of discarded AN husks can not only achieve efficient use of resources but also provide new directions and economic value for the development of AN industry.

So, how can we promote the sustainable development of the AN industry? Perhaps optimizing the extraction process of areca alkaloids in AN husk may be one of the answers. The traditional methods for extracting alkaloids generally involve the use of organic solvents such as ethanol, ether or chloroform, and the development of innovative extraction techniques has also significantly improved the extraction efficiency, reduced the extraction time and reduced the use of organic solvents [22,23]. Among them, subcritical water extraction (SWE) has captured more and more attention due to its safety, efficiency and environmental protection [24]. Subcritical water is defined as hot water whose temperature is between 100 °C and 374 °C under the critical pressure (1–22.1 MPa), which is enough to maintain its liquid state. As the temperature rises, the dielectric constant, viscosity, and surface tension of water decrease steadily, while its diffusion coefficient increases. When the temperature reaches 250 °C and the pressure is 25 bar, the dielectric constant drops to 25, which is between the dielectric constants of methanol (ε = 33) and ethanol (ε = 24) at 25 °C. This gives water properties similar to organic solvents, enabling it to dissolve various moderately polar and low-polar compounds [25]. It is precisely because of this unique property of subcritical water that it can be used as an extraction solvent in line with the principles of green chemistry. Therefore, SWE was usually used in the food and pharmaceutical industries to extract bioactive ingredients, such as polysaccharides, proteins, antioxidants, and polyphenols, which could maintain their biological activity and stability during the extraction process [26,27].

Up to now, the main extraction methods applied for areca alkaloids include solvent extraction [28], heat reflux extraction [29], ultrasonic-assisted extraction [30], supercritical CO_2_ extraction [31] and SWE extraction [32]. As shown in Table 1, five methods were used to extract areca alkaloids from different parts of AN, resulting in varying extraction rates. Among them, HPLC was applied to determine the content of arecoline monomer in Chen’s work, Zhan’s work, Luo’s work and Wu’s work. The highest arecoline extraction rate was achieved in Chen’s work, reaching (8.330 ± 0.109) mg/g. However, hydrochloric acid was required in this extraction method, which caused certain pollution. In Kang’s work and this work, the total content of areca alkaloids was detected by UV–Vis after SWE treatment in AN seed and husk, respectively. Since the extraction object of Kang’s work and this work is different, it is not appropriate to compare those that use the subcritical water extraction process. However, for the same extraction object, the differences between extraction methods are more evident. For example, SWE extraction is superior to ultrasonic-assisted extraction and supercritical CO_2_ extraction. In summary, subcritical water can be considered as an effective extraction method for areca alkaloids, but further exploration is still needed for industrialization.

In the context of our continuous interest [33,34,35], we are curious about the total alkaloid content of AN husk with the aid of SWE. As expected, the optimal SWE process for the total alkaloid content from AN husk was obtained in this work. Furthermore, we also explored a preliminary modification effect of this SWE process on AN husk’s fibers. We hope that this paper has also contributed by providing insights into SWE’s influence on the structure of AN husk.

## 2. Results and Discussion

### 2.1. The Standard Curve of Areca Alkaloids

**Figure 1:**
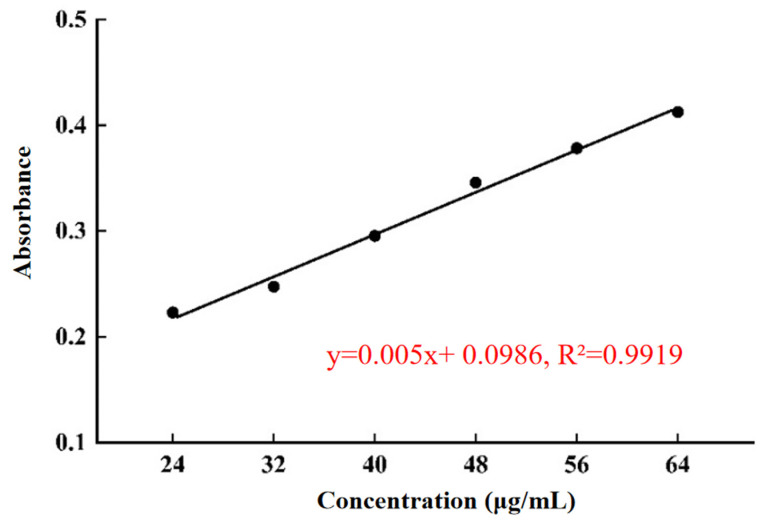
The standard curve of areca alkaloids.

Following the method described in Section 3.2, plot the standard curve of areca alkaloids with its concentration (μg/mL) on the x-axis and the absorbance on the y-axis (Figure 1). The linear regression equation for this standard curve is calculated as y = 0.005x + 0.0986, R^2^ = 0.9919.

### 2.2. Single-Factor Influence on the Extraction Rate of Areca Alkaloids

#### 2.2.1. The Influence of Extraction Time

**Figure 2:**
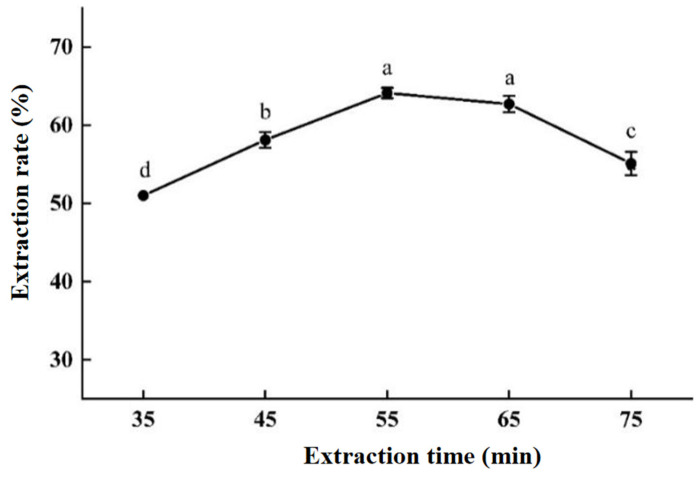
The influence of extraction time. The “a–d” marking method in SPSS difference analysis (*p* < 0.05): any with the same marker letter indicate no significant difference, while those with different marker letters indicate significant differences.

As shown in Figure 2, within a certain timeframe, the extraction rate of areca alkaloids increases with the passage of extraction time (35~55 min). However, it gradually decreases from 55 min to 75 min. Overall, the influence of extraction time on the extraction rate of areca alkaloids shows a trend of first increasing and then decreasing. This is similar to the result obtained by Kang’s group [32], that as the extraction time is prolonged, the extraction rate of areca alkaloids in AN seed first increases and then decreases, and its peak time is 45 min. When the extraction time is 55 min, although the extraction rate of areca alkaloids reaches its peak, there is no significant difference compared to the results at an extraction time of 65 min, suggesting that the actual peak may exist between 55 and 65 min. The reason might be that subcritical water has a strong dissolution and decomposition ability under high temperature and pressure conditions, which could finish the extraction in a short time. If the extraction time is too short, the areca alkaloids might not be fully dissolved. If the extraction time is too long, the structure of areca alkaloids might be destroyed, thus reducing the extraction rate. Therefore, 55 min should be chosen as the optimal extraction time.

#### 2.2.2. The Influence of Liquid-to-Solid Ratio

**Figure 3:**
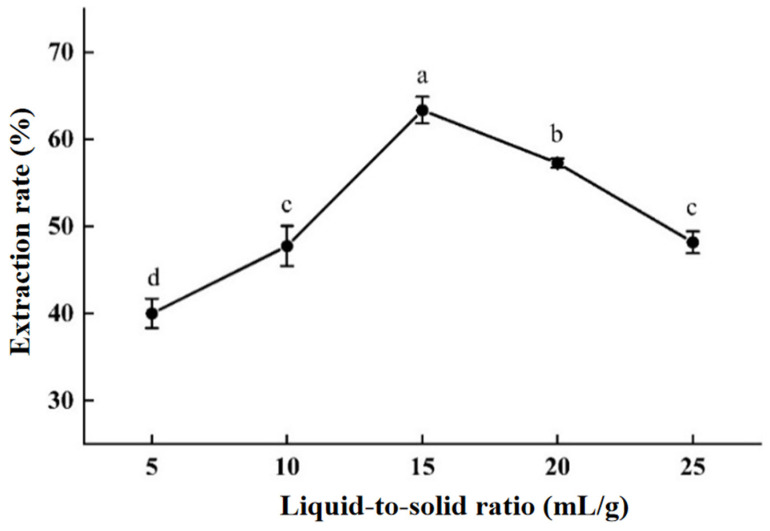
The influence of liquid-to-solid ratio. The “a–d” marking method in SPSS difference analysis (*p* < 0.05): any with the same marker letter indicate no significant difference, while those with different marker letters indicate significant differences.

As shown in Figure 3, when the liquid-to-solid ratio is small, the extraction rate of areca alkaloids is positively correlated with it. Unlike the optimal liquid-to-solid ratio (40:1) obtained in Kang’s work [32], it is found that a liquid-to-solid ratio of 15:1 could reach the peak in this work, which has a significant difference from the results obtained under other conditions, and greatly saves on extraction cost. After reaching the peak, as the liquid-to-solid ratio increases, the extraction rate of areca alkaloids decreases. The possible reason is that the lower liquid-to-solid ratio means a smaller contact area between solvent and sample, which negatively affects the dissolution rate, making it unfavorable for the extraction. However, an excessively high liquid-to-solid ratio may affect the heat transfer of the entire extraction system, leading to a decrease in the extraction rate of areca alkaloids. Therefore, a liquid-to-solid ratio of 15:1 should be chosen for the subsequent experiments.

#### 2.2.3. The Influence of Extraction Temperature

**Figure 4:**
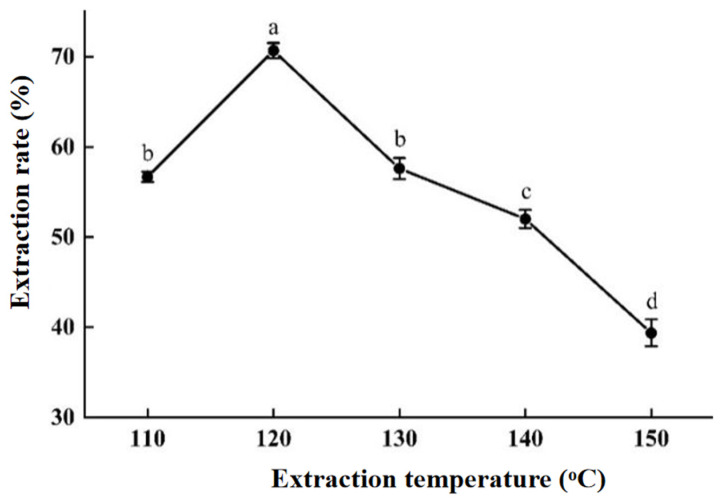
The influence of extraction temperature. The “a–d” marking method in SPSS difference analysis (*p* < 0.05): any with the same marker letter indicate no significant difference, while those with different marker letters indicate significant differences.

As mentioned in the Introduction section, the subcritical water demonstrated similar properties to organic solvents, enabling it to dissolve various moderately polar and low-polar compounds; an exploration of the influence of extraction temperature is conducted here. As shown in Figure 4, the extraction rate of areca alkaloids at 120 °C reaches its maximum value and has a significant difference from the other groups. Compared with Kang’s optimal extraction temperature of 140 °C for areca alkaloids in AN seed [32], our optimal arecoline extraction temperature is lower, which undoubtedly saves more energy. As the extraction temperature increases, the polarity of subcritical water decreases, and the diffusion speed and mass transfer rate increase, allowing it to rapidly penetrate into the AN matrix. It results in an increased contact area between the subcritical water and the sample, leading to a rapid rise in the extraction rate of areca alkaloids. But when the extraction temperature exceeds 120 °C, the extraction rate of areca alkaloids begins to decline rapidly, perhaps due to the destroyed structure of areca alkaloids or other side reactions under high temperatures [36]. Therefore, the optimal extraction temperature is 120 °C in this section.

### 2.3. The Response Surface Methodology (RSM) Optimization

#### 2.3.1. The Interaction Effects Between Factors

According to the above results provided by the single-factor experiments, the optimal extraction rate of areca alkaloids is observed at an extraction time of 55 min, a liquid-to-material ratio of 15:1, and an extraction temperature of 120 °C, respectively. Moreover, the influence of each variable is such that too much is detrimental; excessively long extraction times, overly high liquid-to-material ratios, or excessively high extraction temperatures all have negative impacts. To further study the interaction effects among factors, three main factors that have greater impact on the yield of areca alkaloids are selected for Box–Behnken central composite experimental design [37]. The experimental results are shown in Table 2. Based on these results, a regression equation for the extraction rate of areca alkaloids (Y) as a function of extraction temperature (A), liquid-to-solid ratio (B) and extraction time (C) was established: Y = 67.10 + 9.44A + 1.26B − 13.13C − 8.40AB + 10.72AC + 1.93BC + 1.98A^2^ − 4.82B^2^ + 1.00C^2^.

Further analysis of variance found that the regression model’s *F*-value is 5.3, which is greater than F_0_._05_ (9,5) = 0.2872, with *p* = 0.0405 < 0.05, indicating that the model variance is significant and can adequately explain the variation in the data, which is of great importance for optimizing the SWE process. Secondly, the lack of fit has a *p*-value of 0.0705 (>0.05), indicating that there is no significant lack of fit, and the model fits well and provides a certain degree of reliability in predicting the extraction rate of areca alkaloids by SWE. According to these results, we also found that the factor C in this regression model has the most significant effect on the extraction rate of areca alkaloids, followed by factors A and AC. By comparing the *F*-values of each factor in Table 3, its significance order is as follows: extraction time > extraction temperature > liquid-to-solid ratio.

Through reliability analysis in Table 4, it can be seen that the R^2^ of this model is 0.9051, which means that the model can explain 90.51% of the variance in the response surface value (Y), indicating a good fit of the equation and its ability to adequately reflect the influence of various factors on the response value. The coefficient of variation (CV) is an indicator used to measure the degree of variation in sample data. The CV in this model is 11.94%, a relatively low value indicating a relatively small degree of variation in the sample data, suggesting that the experiment was quite precise. In regression models, the Adeq precision is the ratio of the signal (variation in the predicted variable) to the noise (variation in the residuals), and it is generally considered reasonable if it is higher than 4. In this experiment, the Adeq precision value is 7.399, indicating that the signal strength in this model is high, and this model can be used to guide experimental design.

#### 2.3.2. RSM Analysis

**Figure 5:**
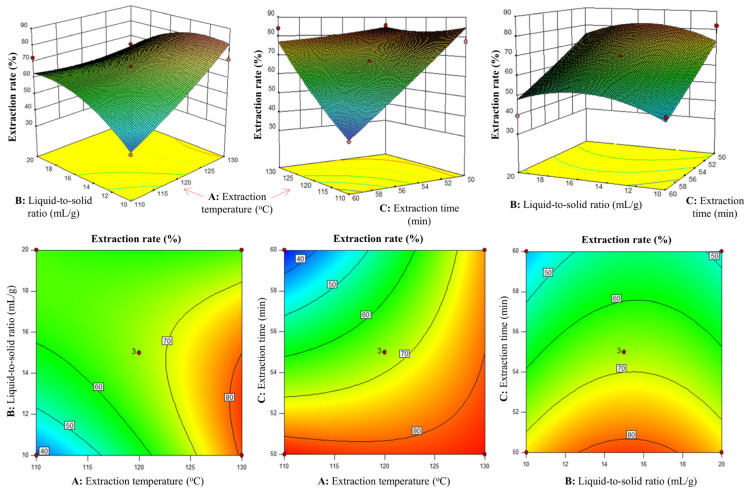
Response surface plot of the interaction between A (extraction time), B (liquid-to-solid ratio) and C (extraction temperature).

Response surface plots and contour plots can visually demonstrate the interaction between various factors. The steeper the response surface or the flatter the contour lines, the stronger the interaction between two factors. By analyzing the results in conjunction with variance analysis, it can be seen from Figure 5 that the response surfaces of both A (extraction time) and B (liquid-to-solid ratio), and B (liquid-to-solid ratio) and C (extraction temperature) are relatively gentle, with no significant mutual influence (*p* > 0.05); compared to the above two groups, the response surface slope obtained from the interaction between A (extraction temperature) and C (extraction time) is the steepest, and its contour lines are also the flattest among the three groups, indicating the strongest interaction between the two, which reaches a significant level (*p* < 0.05).

#### 2.3.3. Prediction and Verification of Optimal Conditions

According to the prediction of the regression model, the optimal parameters for SWE of areca alkaloids are as follows: extraction temperature 110.87 °C, liquid-to-solid ratio 18.98:1, extraction time 50.01 min, with a predicted extraction rate of 87.27% (8.727 mg/g). Considering practical conditions, the parameters were adjusted to an extraction temperature of 110 °C, liquid-to-solid ratio of 19:1, and extraction time of 50 min. The measured extraction rate was 81.7% (8.17 mg/g), which is close to the predicted value, indicating that the extraction process optimized by RSM experiments is feasible.

### 2.4. The Influence of SWE on AN Husk

#### 2.4.1. X-Ray Diffraction (XRD) Measurement

**Figure 6:**
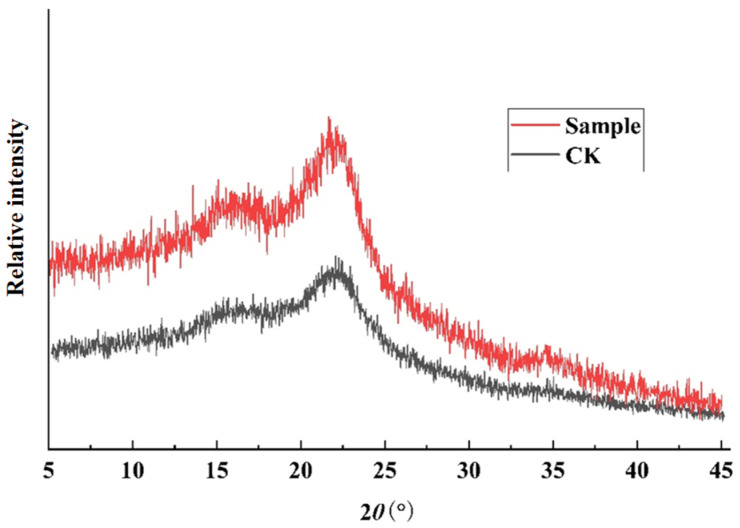
The influence of SWE on the XRD of AN husk.

The influence of SWE on the quality of AN husk was evaluated through XRD. Generally, the crystalline region is mainly contributed by cellulose, while the amorphous region (non-crystalline region) is primarily contributed by lignin, hemicellulose, and amorphous cellulose [38]. Cellulose in AN husk contains both crystalline regions and amorphous regions composed of lignin and hemicellulose. Both the control group (CK) and the SWE treatment group (Sample) showed two broad peaks at 2θ angles of 16° and 22°, which are typically attributed to Type I cellulose [39]. As shown in Figure 6, the peak shapes of the Sample and CK are essentially consistent, with no shift in the two peaks (2θ = 16° and 2θ = 22°), indicating that the crystal type of fibers in AN husk did not change after SWE treatment.

However, as seen in Table 5, compared to the crystallization index of CK (26.5%), the crystallization index of AN husk after SWE treatment for areca alkaloids decreased to 11.4%, a reduction of about 57%. This may be due to the breaking of glycosidic bonds in cellulose under high-temperature conditions. The principle is that under high-temperature conditions, water molecules diffuse into the cellulose, causing its hydrogen bonds to break. As cellulose gradually begins to dissolve, it reacts hydrolytically with water molecules in the system, leading to the breaking of glycosidic bonds [40].

#### 2.4.2. Fourier Transform Infrared Spectroscopy (FT-IR) Measurement

**Figure 7:**
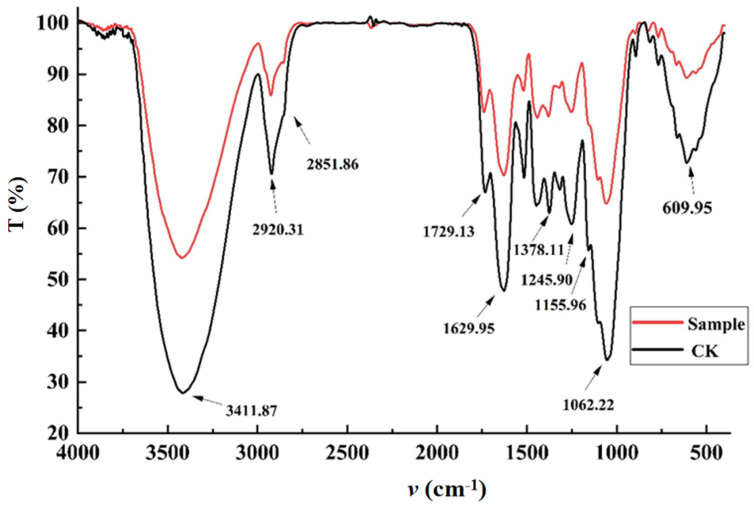
The influence of SWE on FT-IR spectrum of AN husk.

FT-IR utilizes Fourier transformation to decompose complex wave forms in infrared spectra into a series of sinusoidal waves of basic frequencies, thereby obtaining information on the vibrations of different chemical bonds. By analyzing and comparing these pieces of information, the chemical composition and structure of the sample can be determined. As shown in Figure 7, the high-intensity broad absorption peak at 3411.87 cm^−1^ represents the stretching vibration peak of associated O-H, which mainly originates from polyphenols and polysaccharides in AN husk. The characteristic absorption peaks at 2920.31 cm^−1^ and 1378.11 cm^−1^ correspond to -CH_3_, while the peak at 2851.86 cm^−1^ corresponds to the stretching vibration peak between carbon and hydrogen atoms in -CH_2_, reflecting the basic structure of carbohydrates. The main source may probably be the cellulose in AN husk. The strong peak at 1629.95 cm^−1^ reflects -C=C-, which possibly mainly originates from areca alkaloids and unsaturated fatty acids in AN husk. Multiple sharp absorption bands appeared in the range of 625–1365 cm^−^, which are speculated to be the skeletal vibration bands of aromatic rings and mainly originate from polyphenols in AN husk. When an alkene double bond is connected to the carbon atom of C=O, the conjugation effect causes the vibration frequency of the lipid carbonyl group to shift to lower frequencies. Therefore, the strong peak at 1729.13 cm^−^ reflects the C=O structure, and the absorption peaks at 1245.90 cm^−^ and 1155.96 cm^−^ indicate the presence of C-O. From this, it can be inferred that there is an ester group, which primarily originates from the fats in AN husk. And the peaks generated at 1062.22 cm^−^ and 609.95 cm^−^ are consistent with the characteristic peaks in the infrared fingerprint spectrum of AN obtained by Wang’s group [41].

Compared to the CK, after SWE treatment, the infrared spectral absorption intensity of AN husk generally decreased. According to Lambert’s law, the height of peaks in an infrared spectrum is directly proportional to the amount of related functional groups. To some extent, the height of peaks can reflect the content of a certain component in the sample. For example, the characteristic peak of -C=C- at 1629.95 cm^−1^ significantly decreased compared to the CK, which may be due to the fact that while extracting areca alkaloids through SWE treatment, components containing carbon–carbon double bonds such as arecaidine and demethylarecoline were also extracted. In addition, this could also be due to the high temperature and pressure conditions during SWE, causing the double bonds to break.

## 3. Materials and Methods

### 3.1. Materials

The fresh AN was purchased at a Hainan betel nut plantation in May 2022. All other chemicals, including acetic acid, sodium acetate, chloroform, anhydrous sodium sulfate, potassium hydroxide, anhydrous ethanol, bromocresol green and potassium bromide, were purchased from Guangzhou Chemical Reagent Factory. And the arecoline hydrobromide, as a standard sample, was purchased from Sigma-Aldrich (Shanghai) Trading Co., Ltd. (Shanghai, China).

### 3.2. Sample Preparation

After removing the AN kernel, the fresh AN husk was sliced, placed in a hot air-drying oven to be dried at 50 °C, pulverized by a universal crusher, sieved through a 40-mesh sieve, and then put into a drying bottle for later use. The AN husk was processed by Subcritical Water Extraction Test Device (CBEW-5L) from Henan Sub-critical Extraction Technology Research Institute Co., Ltd. (Anyang, China), and the corresponding extract sample in this study was provided by this company.

### 3.3. SWE Process Parameters

During the pressurized extraction process of the subcritical extraction unit, an inert gas (nitrogen) is introduced from the outside, and the preset test pressure in the tank is controlled by the exhaust valve. The stirring speed is set at 350 rpm during the extraction process, and the extraction temperature and time are set according to the designed temperature and time of the experiment.

### 3.4. Methods

#### 3.4.1. SWE Method

Weigh a certain mass of ANs and measure an appropriate amount of distilled water to achieve the specified liquid-to-solid ratio (distilled water: AN (*v*/*w*)). Add to the SWE apparatus and extract according to the experimental design parameters for extraction time, liquid-to-solid ratio, and extraction temperature. After centrifuging the extract, take the supernatant for future use.

#### 3.4.2. Determination and Calculation of Extraction Rate of Areca Alkaloids

The UV–Vis spectrophotometry was recorded on Presee TU-1810 (Beijing, China). Employ UV–Vis data to determine the content of areca alkaloids [42]. After plotting the standard curve for hydrobromic acid salt of areca alkaloids, set *λ* = 618 nm to measure absorbance, then substitute into the standard curve to calculate the sample concentration. The formula for calculating the extraction rate of areca alkaloids is:

$$Y=\frac{n\times V\times c}{M}\times 100\%$$

where Y is the extraction rate of areca alkaloids (%); n is the dilution factor; M is the sample mass (g); V is the volume for constant volume (mL); c is the concentration of areca alkaloids (μg/mL).

#### 3.4.3. Single-Factor Experiments

(1) The effect of different extraction times on extraction efficiency

Set the pressure at 2.5 MPa, SWE temperature at 120 °C, and liquid-to-solid ratio (*v*/*w*~distilled water:AN) at 15:1 (mL/g). Set the subcritical treatment times to 35, 45, 55, 65, and 75 min, with three replicate experiments.

(2) The effect of different liquid-to-solid ratios on extraction efficiency

Set the pressure at 2.5 MPa, subcritical extraction time at 55 min, and SWE temperature at 120 °C. Set the liquid-to-solid ratios to 5:1, 10:1, 15:1, 20:1, and 25:1 (mL/g), with three replicate experiments.

(3) The effect of different extraction temperatures on extraction efficiency

Set the pressure at 2.5 MPa, extraction time at 55 min, and liquid-to-solid ratio at 15:1 (mL/g). Increase the SWE temperature by 10 °C each time, ranging from 110 °C to 150 °C, with three replicate experiments.

#### 3.4.4. RSM Optimization

Based on the above experiments, design a three-factor, three-level response surface optimization experiment for the SWE method to determine the optimal conditions for extracting areca alkaloids. The experimental factors and levels are shown in Table 6.

#### 3.4.5. Analysis of the SWE’s Effect on AN Structure

(1) XRD measurement

The XRD data were collected by Bruker D8 Advance (Bruker, Billerica, MA, USA). The detection conditions for XRD were as follows. At room temperature, set the voltage to 40 kV and the current to 100 mA, with a transition time of 1 s [43]. Scan the sample within the diffraction angle (2θ) range of 10°~70° with a scanning step size of 0.02° to obtain the XRD pattern. Use the peak intensity method to calculate the crystallization index (CI) of the sample. The CI is calculated using Segal’s method, as given below [44]:

$$CI=\frac{I_{002}-I_{am}}{I_{002}}\times 100\%$$

where I_002_ is the maximum intensity of the 002 lattice diffraction angle; I_am_ is the diffraction intensity of the amorphous region.

(2) FT-IR measurement

The FT-IR data were recorded on Bruker Tensor 27 FTIR. Mix the sample powder with KBr powder at a ratio of 1:10 (*w*/*w*) in an agate mortar, grind it, and then press it into tablets using a tablet press. Scan within the wavelength range of 500~4000 cm^−1^ with a resolution of 2 cm^−1^ to obtain the Fourier transform infrared spectrum [45].

#### 3.4.6. Data Analysis

Each group of experiments is measured in triplicate, and data are analyzed using SPSS 26.0 statistical software with a significance level of 0.05. Design-Expert 13.0.5 software is used for response surface experimental data analysis.

## 4. Conclusions

In this study, we obtained the optimal process parameters of SWE for areca alkaloids through the single-factor experiments and RSM optimization as follows: extraction temperature of 110.87 °C, liquid-to-solid ratio of 18.98:1 and extraction time of 50.01 min. The factors influencing the process in order of significance were extraction time > extraction temperature > liquid-to-solid ratio. Considering practical conditions, the parameters were adjusted to the extraction temperature of 110 °C, the liquid-to-solid ratio of 19:1 and the extraction time of 50 min. The measured extraction rate was 81.7%, which was close to the predicted value, indicating that the extraction process optimized by RSM is feasible. The XRD measurement after SWE treatment indicated that the crystal type of AN husk fibers did not change, and the CI of AN husk decreased. The FT-IR measurement after SWE treatment revealed that the infrared spectral absorption intensity of AN husk generally decreased. The preliminary XRD and FT-IR results showed that SWE had a significant influence on the structure of AN husk, possibly damaging part of the crystalline regions of cellulose in AN husk or reducing the concentration of various functional groups.

This work, starting from the perspective of food safety for AN husk, optimized the optimal SWE for areca alkaloids and further verified the modifying influence of SWE on crude fibers in AN husk. This provides certain data references for subsequent studies on utilizing SWE for processing AN husk. However, this experiment only stayed at the theoretical level for research on the softening of AN husk fibers. Therefore, sensory analysis and texture characteristic testing or other more intuitive experiments can be carried out on betel nuts treated with subcritical water afterwards, so as to further verify the actual effect of subcritical water on softening AN husk fibers. Further exploration is still needed for industrialization.

## Figures and Tables

**Table 1:** Five extraction methods for areca alkaloids.

Extraction Method	Extract Object	Optimal Parameters	Extraction Rate (mg/g)
solvent extraction (Chen’s work) [28]	arecoline in AN waste	the particle size is 60 mesh, the liquid-to-solid ratio is 15:1, the extraction time is 45 min, the extraction time is 2, the extraction temperature is 80 °C, the mass concentration of hydrochloric acid is 0.22%.	8.330 ± 0.109
heat reflux extraction (Zhan’s work) [29]	arecoline in AN inflorescence	the liquid-to-solid ratio is 80:1, the extraction time is 25 min, the extraction time is 3.	4.31 ± 0.047
ultrasonic-assisted extraction (Luo’s work) [30]	arecoline in AN husk	the concentration of ethanol is 85%, the liquid-to-solid ratio is 8:1, the extraction temperature is 65 °C, the pH is 8.	1.987
supercritical CO_2_ extraction (Wu’s work) [31]	arecoline in AN husk	the extraction pressure is 33 MPa, the extraction temperature is 63 °C, the microwave power is 480 W.	4.48 + 0.07
SWE extraction (Kang’s work) [32]	areca alkaloids in AN seed	the extraction time is 43 min, the extraction temperature is 143 °C, the liquid-to-solid ratio is 35:1.	4.23
SWE extraction (This work)	areca alkaloids in AN husk	the extraction temperature is 110 °C, the liquid-to-solid ratio is 19:1, the extraction time is 50 min.	8.17

**Table 2:** The experimental results for further study.

Entry	A (°C)	B (mL/g)	C (min)	Y (%)
1	110	10:1	55	47.2
2	110	20:1	55	72.4
3	130	10:1	55	72.9
4	130	20:1	55	64.5
5	120	10:1	50	81.9
6	120	10:1	60	48.0
7	120	20:1	50	74.7
8	120	20:1	60	48.5
9	110	15:1	50	77.6
10	130	15:1	50	85.0
11	110	15:1	60	33.7
12	130	15:1	60	84.0
13	120	15:1	55	64.0
14	120	15:1	55	69.1
15	120	15:1	55	68.2

**Table 3:** Analysis of variance table for regression model.

Source	Sum of Squares	Degree of Freedom	Mean Square	*F*-Value	*p*-Value	Significant
Model	2972.38	9	330.26	5.3	0.0405	*
A	712.53	1	712.53	11.43	0.0197	*
B	12.75	1	12.75	0.2045	0.67	-
C	1378.12	1	1378.12	22.1	0.0053	**
AB	282.24	1	282.24	4.53	0.0867	-
AC	460.1	1	460.1	7.38	0.0419	*
BC	14.82	1	14.82	0.2377	0.6465	-
A^2^	14.4	1	14.4	0.231	0.6511	-
B^2^	85.96	1	85.96	1.38	0.2932	-
C^2^	3.69	1	3.69	0.0592	0.8174	-
Residual	311.73	5	62.35	-	-	-
Lack of fit	296.91	3	98.97	13.36	0.0705	-
Pure error	14.82	2	7.41	-	-	-
Total	3284.12	14	-	-	-	-

Note: ** *p* < 0.01, * *p* < 0.05, - *p* > 0.05.

**Table 4:** Reliability analysis of the regression model.

Term	Value
R^2^	0.9051
R_adj_^2^	0.7342
CV	11.94%
Adeq precision	7.399

**Table 5:** The influence of SWE on crystallinity of AN husk.

AN Group	Crystallization Index (%)
CK	26.5
Sample	11.4

**Table 6:** Factors and levels of response surface experiment.

Levels	Factors		
	Extraction Temperature (°C)	Liquid-to-Solid Ratio (mL/g)	Extraction Time (min)
−1	110	10:1	50
0	120	15:1	55
+1	130	20:1	60

## Data Availability

The original contributions presented in this study are included in the article. Further inquiries can be directed to the corresponding authors.
